# Metagenomic Analysis of Upwelling-Affected Brazilian Coastal Seawater Reveals Sequence Domains of Type I PKS and Modular NRPS

**DOI:** 10.3390/ijms161226101

**Published:** 2015-11-27

**Authors:** Rafael R. C. Cuadrat, Juliano C. Cury, Alberto M. R. Dávila

**Affiliations:** 1 Computational and Systems Biology Laboratory, Oswaldo Cruz Institute, Fiocruz, Avenida Brasil 4365, Rio de Janeiro CEP 21040-360, Brazil; rafaelcuadrat@gmail.com; 2 Leibniz-Institute of Freshwater Ecology and Inland Fisheries, Alte Fischerhuette 2, OT Neuglobsow, Stechlin 16775, Germany; 3 Berlin Center for Genomics in Biodiversity Research, Königin-Luise-Straße 6-8, Berlin 14195, Germany; 4 Molecular Microbiology Laboratory, Federal University of São João del-Rei, Sete Lagoas *Campus* Rua Sétimo Moreira Martins 188, Itapoã II, Sete Lagoas CEP 35702-031, Brazil; jccury@hotmail.com

**Keywords:** PKS, NRPS, metagenomics, environmental genomics, upwelling, coastal environment

## Abstract

Marine environments harbor a wide range of microorganisms from the three domains of life. These microorganisms have great potential to enable discovery of new enzymes and bioactive compounds for industrial use. However, only ~1% of microorganisms from the environment can currently be identified through cultured isolates, limiting the discovery of new compounds. To overcome this limitation, a metagenomics approach has been widely adopted for biodiversity studies on samples from marine environments. In this study, we screened metagenomes in order to estimate the potential for new natural compound synthesis mediated by diversity in the Polyketide Synthase (PKS) and Nonribosomal Peptide Synthetase (NRPS) genes. The samples were collected from the Praia dos Anjos (Angel’s Beach) surface water—Arraial do Cabo (Rio de Janeiro state, Brazil), an environment affected by upwelling. In order to evaluate the potential for screening natural products in Arraial do Cabo samples, we used KS (keto-synthase) and C (condensation) domains (from PKS and NRPS, respectively) to build Hidden Markov Models (HMM) models. From both samples, a total of 84 KS and 46 C novel domain sequences were obtained, showing the potential of this environment for the discovery of new genes of biotechnological interest. These domains were classified by phylogenetic analysis and this was the first study conducted to screen PKS and NRPS genes in an upwelling affected sample

## 1. Introduction

Metagenomics has been largely used to unlock the huge biodiversity and biochemical potential from uncultivable organisms [1,2]. Beside the large number of metagenomics studies carried in marine environments, only few studies have explored the marine waters of the Brazilian Coast using metagenomic approaches. Among them, Gregoracci *et al*. [3] studied the bacterioplankton of Guanabara Bay (the second largest bay of Brazil), in the Rio de Janeiro state. Trindade-Silva and colleagues [4] characterized the microbial diversity associated to the marine sponge Arenosclera brasiliensis from water of the João Fernandinho beach (Rio de Janeiro State) [4]. Cury and colleagues studied the taxonomic diversity of coastal seawater from Arraial do Cabo (Rio de Janeiro State, Cabo Frioregion) [5], an important fishing and tourism region influenced by an upwelling system and anthropogenic activity, with sporadic sewage emissions [6,7]. Upwelling is characterized by the up-flow of cold and nutrient-rich waters, disturbing ecosystem dynamics then increasing biomass and primary production of these environments. From the Brazilian coastal waters, it is at Arraial do Cabo that this upwelling effect is most intense [8]. In a previous study from our group, the SSU rDNA amplification approach was used in Arraial do Cabo [5] and limited to the general taxonomic assembly of the microorganisms, rather than providing information about the metabolic potential of the community [9]. By using a whole metagenome pyrosequencing approach it should be possible not only to estimate the biodiversity, but also to explore functional gene diversity and select genes of biotechnological interest [10].

The Polyketide Synthases (PKSs) and Nonribosomal Peptide Synthetases (NRPS) encode two families of secondary metabolite enzymes from microorganisms that are of great interest to the biotechnological industry. They are responsible for the production of a variety of compounds from antibiotics to pigments and from antitumor agents to immunosuppressants [11,12]. There are three types of PKS. The type I PKS genesencode large multi-domain enzymes with all the necessary components for elongating and processing the polyketide chain of the same protein. They can be classified as modular (the most common from bacteria) or iterative (from fungi and bacteria) [13,14]. The type II PKS genes encode multi enzyme complexes (with three or more enzymes) acting in an iterative manner [15]. The type III *PKS* genes encode enzymes responsible for the production of Chalcones in plants and polyhydroxy phenol in bacteria [16]. In comparison, the NRPS genesencode modular enzymes that can activate and condense amino acids to produce small peptides (non-ribosomal peptides) [17]. Most new genes of these families have been discovered in genomes from the phylum Actinobacteria, which accounts for 5% to 10% of total bacteria on marine water [18,19,20]. However, the natural products from the most abundant phylum from marine oligotrophic environments (Proteobacteria) are poorly studied [21]. Most of the previous studies conducted to screen for PKS and NRPSs diversity were performed in soil or host associated (marine invertebrates) microbiomes [22,23]. However, Grossart *et al.* showed in 2004 that many members of Rhodobacteraceae family, isolated from organic aggregates in the Wadden Sea in Germany, produce secondary metabolites (with bacterial inhibitory activity encoded by PKS and NRPS genes) [24,25]. Thus, many antimicrobial peptides were found in marine proteobacteria [26,27,28].

In this study, we screened conserved domains of PKS and NRPS in order to assess the microbes potential from the seawater of Praia dos Anjos (Angel’s Beach), in Arraial do Cabo, Brazil by using HMM profiles. The sample site is close to the one sampled by Cury and colleagues, called POS sample [5] and it was collected during the summer season. We choose this site due its high abundance of Proteobacteria from Rhodobacteraceae family (with great metabolic potential), and the presence of Actinobacteria, which were in greater abundance than at the other sites analyzed in the previous study [5]. The samples were collected during the summer season because the upwelling phenomena in this region occurs with higher frequency and intensity during this season [5,8]. At the time of writing, to our knowledge, this is the first study aiming to find novel PKS and NRPS genes in an upwelling-affected environment.

## 2. Results and Discussion

### 2.1. Metagenomic Reads Assembly

A total of 651,083 and 542,647 reads were obtained for samples P and E, respectively. After pre-processing, this number decreased to 595,534 and 469,354, respectively. The average size of the reads was 588 and 595 base pairs (bp) for P and E samples, respectively.

With the aim of obtaining contigs from the metagenomic reads, we used the CAP3 program [29]. The assembly of environmental sequences is a complex problem and so many algorithms were proposed to address it [30,31,32,33]. The main problems of the assembly are the low coverage and the possible formation of chimeras (especially in environments with high diversity) [34,35]. Using the CAP3 with the very stringent default parameters, we tried to minimize the problem of chimeras, but the low coverage only can be outlined with a more deep sequencing effort. We obtained a total of 29,074 contigs and 269,587 singlets for sample P. The mean size of the contigs was 1368.4 bp (the largest contig had 17,926 bp).

For sample E, we obtained 20,792 contigs and 396,371 singlets. The mean size of the contigs was 1018.2 bp (the largest contig had 9281 bp). From these sequences (singlet + contigs), were also obtained 409,111 and 451,722 ORFs for sample P and E, respectively, using METAGENMARK [36].

### 2.2. Screening of NRPS and PKS Domains

Many studies have been conducted for functional or PCR-based screening of PKS and NRPS in diverse environments [22,23,37,38]. However, despite the growth of metagenomes databases, to date, only few studies were performed using computational approaches to screen PKS in whole shotgun sequenced metagenomes. One of these was conducted by Foerstner *et al.* [39] where six natural environment datasets were screened using a HMM profile approach. Moreover, no study has been performed to screen secondary metabolites genes in water from upwelling-affected coastal environment.

Due to the high abundance of *Roseobacter* clade organisms and Cyanobacteria in the samples from Arraial do Cabo (data not shown), we decided to conduct an *in silico* screening of PKS and NRPS genes. The aim of this study was to evaluate the potential of the studied environment to provide new secondary metabolites, since many studies have shown the presence of these genes in the genomes of these taxonomic groups (*Roseobacter* clade organisms and Cyanobacteria) [24,26,27,28].

#### 2.2.1. KS Domain

In this study, two KS pHMM were built: from sequences of modular KS and iterative KS. These pHHM were used to retrieve putative KS domains in metagenomes from Arraial de Cabo and the NapDos was used because this system has the capacity to classify KS and C sequences from poorly assembled genomes and metagenomes [40]. Using both pHMMs, a total of 102 KS sequences were obtained. Using the KS modular pHMM, we found a total of 28 hits in sample P and 37 in sample E. These sequences were submitted to BLASTP against RefSeq protein database (*E*-value cutoff e^−5^). Only seven sequences returned no hits, one from sample P and six from sample E. Using the annotation of the five best hits from RefSeq database it was possible to confirm the PKS annotation of 13 sequences (46.42%) and six (72.97%) of the KS sequences of P and E samples, respectively.

All KS sequences were submitted to NapDoS [40]. For sample P, it was possible to classify 23 (78.57%) sequences, whereas for sample E, it was possible to classify 34 (91.89%) sequences.

Using the KS iterative, we obtained 21 and 16 sequences from sample P and sample E, respectively. These sequences were submitted to BLASTP against RefSeq Protein (*E*-value cutoff e^−5^). For sample P, all the sequences showed hits on BLASTP, and for sample E, only two sequences did not show hits. Using the annotation of the five best hits from RefSeq database, it was possible to confirm the PKS annotation of 28.57% (6/21) and 56.25% (9/16) of the sequences from samples P and E, respectively. These sequences were also submitted to NapDoS. For sample P, it was possible to classify 71.42% (15/21) of the sequences, whereas for sample E, it was possible to classify 75% (12/16) of the sequences. From the total KS domains obtained by both pHMM (102), 38 sequences from sample P and 46 sequences from sample E (totalizing 84 sequences—82.35%) were confirmed *in silico* as KS (by blastP and/or NapDos results), respectively. The false positives found (17.65%) were expected, because the HMM approach is very sensitive for the detection of distant homologs [41] and the Fatty Acid Synthase (FAS) is homologous to PKS [42]. The advantage of the pHMM usage to screen type I PKS in metagenomic shotgun data and the possible recovery of false positives was discussed on a study performed by Foerstner *et al*. [39]. Table 1 summarizes the results of these analyses.

From the 84 type I KS (confirmed by Blast and/or NapDos), four were similar to Rhodobacteraceae organisms in the BLASTP results (against RefSeq protein). From these four KS sequences, two were classified in the phylogenetic tree as hybrid KS/PKS enzymes, agreeing with results obtained by Martens *et al*. [25], showing many hybrid enzymes in isolates from the *Roseobacter* clade.

The relative abundance of KS domains present in water was 0.0092% (38/409,111) from sample P and 0.0101% (46/451,722) from sample E. In the study of Foerstner *et al.* [39], the environment with the higher KS domain abundance was Minnesota farm soil [43], where 52 type I KS were found in 183,536 ORFs (0.0283%), 2.8 times more abundant than sample E of the present study. Soil environments commonly possess a high diversity of secondary metabolites because the microorganisms compete intensely with each other [44]. In addition, in the same study, the samples from an open ocean oligotrophic region (Sargasso Sea) [45] (ranging from 0.1 to 0.8 μm like sample P), were screened, showing 69 type I KS sequences in 1,214,207 ORFs (0.0056%), a relative smaller abundance than our sample P. These results confirm the potential of the costal upwelling-affected metagenome for the screening of secondary metabolites.

**Figure 1:**
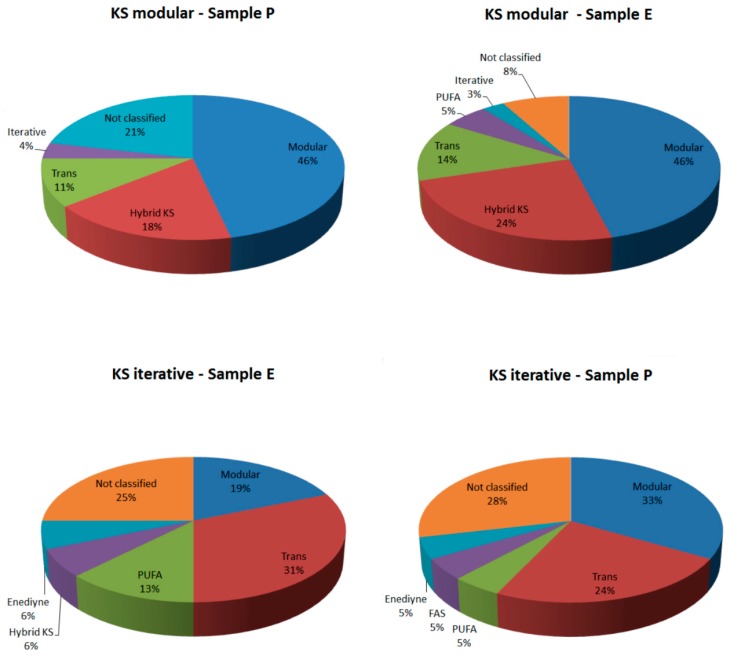
Classification of KS domain by NapDos. The sequences wereobtained with pHMM KS modular and pHMM KS iterative, from both samples.

Figure 1 shows the classification of KS domains obtained from both samples using both pHMMs.

**Table 1:** The total of hits obtained by KS pHMMs (modular and iterative) for sample P and E. In addition, the table shows the total of sequences with annotation confirmed by Blast (five best hits) and the total of sequences classified by NapDos.

Sample	KS Modular (Total Hits)	KS Iterative (Total Hits)	KS Modular (Confirmed by Blast)	KS Iterative (Confirmed by Blast)	KS Modular (Classified by NapDos)	KS Iterative (Classified by NapDos)
Sample P	28	21	13 (46.42%)	6 (28.57%)	23 (78.57%)	15 (71.42%)
Sample E	37	16	27 (72.97%)	9 (56.25%)	34 (91.89%)	12 (75%)
Total	65	37	40	15	57	27

**Figure 2:**
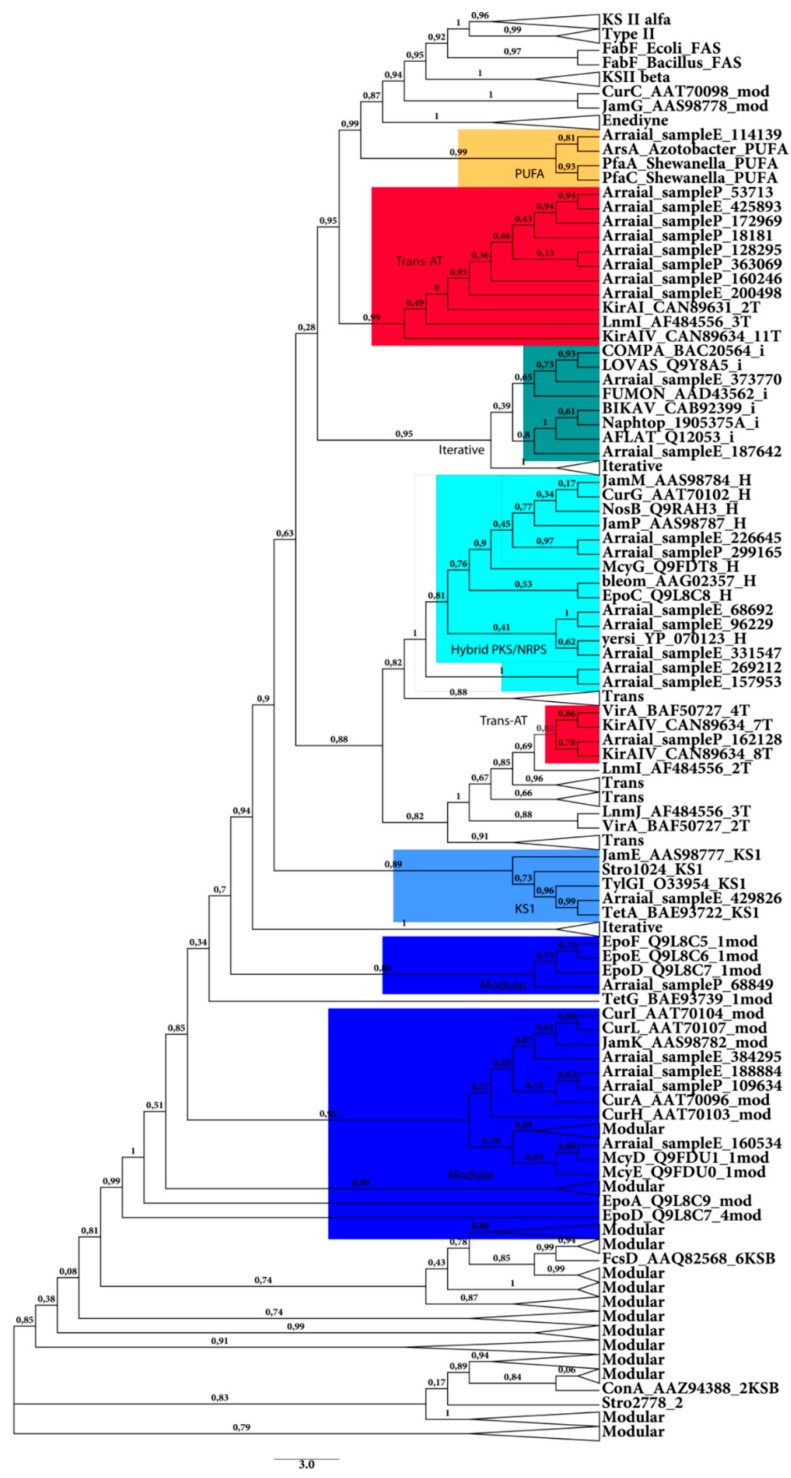
Phylogenetic tree of environmental KS domains (larger than 200 amino acids) obtained from both sample (by KS modular and iterative pHMMs) with reference NapDoS sequences. The tree was generated by NapDos pipeline (using FASTTREE and Maximum Likelihood algorithm). Confidence values are showed on nodes. The shadows show the different groups (Modular, KS1, Trans, Hybrid, iterative and PUFA).

Most of the sequences retrieved with modular KS pHMM were classified as modular by NapDoS (46% from both samples) and Hybrid KS (18% from sample E and 24% from sample P). These hybrid domains can be modular or iterative and are present on hybrid PKS/NRPS enzymes [46]. The Trans-AT domain was also present (11% on sample E and 14% on sample P) and, unexpectedly, using this pHMM it proved possible to retrieve some iterative KS domains. On the other hand, the iterative KS pHMM was able to retrieve only a few iterative sequences (only the iterative Enediyne sequences were retrieved). Most of the sequences retrieved with this pHMM on sample E were Trans-AT domains (31%). This result may be due to a bias in the iterative KS pHMM or due to the high abundance of hybrid PKS in this sample because trans-AT domains are common in hybrid systems. However, on sample P, the most abundant type of KS domains retrieved with the iterative KS pHMM was modular. All the sequences classified as polyunsaturated fatty acids (PUFA) by NapDos were manually verified and the best blast hits (against RefSeq) show similarity with PKS domain (with more significant *E*-value than the NapDos result). The high abundance of the KS module can be explained by the fact that in modular PKS the number of copies of each domain is much higher than in iterative PKS [42].

Additionally, aiming for a most accurate classification of the KS sequences, a phylogenetic analysis was performed using the 10 KS sequences from sample P and 15 from sample E (larger than 200 amino acids) to generate a phylogenetic tree on NapDos with the reference sequences (similar to the environmental sequences) (Figure 2).

The topology of the tree corroborates the results from many phylogenetic studies of the KS domain [37,40]. It was possible to separate the homologous Fab (from Fatty acid synthase), the type II KS alpha and beta domains, the polyunsaturated fatty acids (PUFA) domains (with one sequence from sample E in this clade), two clades from the Modular Trans-AT domains (where seven sequences from sample P and two from sample E were classified), the iterative KS (with two sequences from sample E), the hybrid KS (PKS/NRPS hybrid enzymes), with one sequence from sample P and six from sample E, the KS1 domains (typical starter KS KSQ) domains and KS domains in Curacin and Salinisporamide biosynthesis pathways, with one sequence from sample E, and finally the Modular KS domains (where one sequence from sample P and three from sample E were classified). As the results display in Figure 1, the phylogenetic tree shows most of sequences close to modular (Cis, Trans and Hybrid) KS sequences.

It is important to note that only sequences from Sample E were clustered in the clade of iterative KS domains. These results can be explained by the fact that iterative PKS are common in fungi genomes, and sample E is rich in eukaryotes due to the filtration membrane used (0.8 μm).

The discovery of these new KS domains in Arraial do Cabo can lead us to design new primers for PCR screening in a fosmid library and consequently can also lead to the discovery of new active compounds. The relative abundance of KS domains found in the current study confirms that the tropical coastal upwelling-affected environments, normally neglected in these studies, should be also screened for new polyketide compounds.

#### 2.2.2. C Domain

Using the C domain pHMM, a total of 50 hits were obtained, 14 from sample P and 36 from sample E. These sequences were submitted to BLASTP against RefSeq protein (*E*-value cutoff e^−5^). Sample P yielded 11 hits (78.57%) with confirmed annotations. Sample E yielded 32 hits (88.88%) and it was possible to confirm the annotation from 31 of these. All 43 annotated sequences were submitted to NapDos and classified. It was not possible to confirm the annotation of one sequence (7.14%) from sample P and three sequences (8.33%) from sample E by these methods. These results show that our C pHMM model is specific, with low false positive detection.

**Figure 3:**
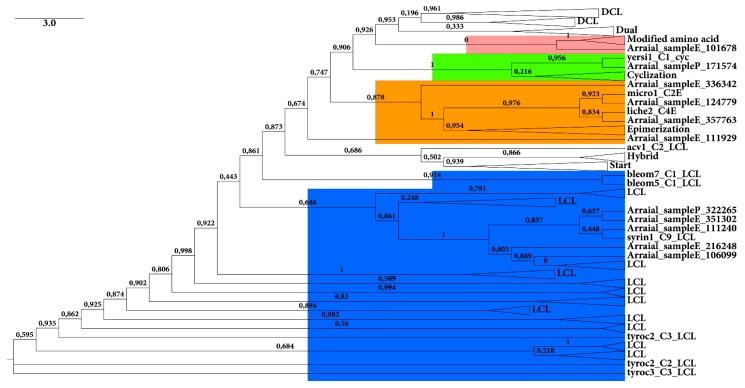
Phylogenetic tree of environmental C domains (larger than 200 amino acids) obtained from both sample (by C domain pHMM) with reference NapDoS sequences. The tree was generated by NapDos pipeline (using FASTTREE and Maximum Likelihood algorithm). Confidence values are shown on nodes. The shadows show the different groups of C domains.

The environmental C domain sequences larger than 200 amino acids were also submitted to phylogenetic analysis using the NapDoS pipeline, with the reference sequences (similar to the environmental sequences). Figure 3 shows the result of this analysis.

The topology of the inferred tree was expected, separating the types of C domains as shown in previous studies [40]. Most of the sequences were from LCL type (four from sample E and onefrom sample P) and Epimerization (four from sample E). The LCL domain catalyzes formation of a peptide bond between two l-amino acids [40]. The abundance of LCL domains was expected as the LCL domain was proposed to be exclusively from gram-negative bacteria (for example, proteobacteria), which are very abundant in the studied environment (data not showed).

As in the PKS screening, sample E showed a higher abundance of the NRPS C domain than sample P. This may be due to the presence of marine snow in sample E, with competition for space and nutrients in the particle-associated bacteria as a selective force [47].

## 3. Experimental Section

### 3.1. Dataset

The sequences were generated by a previous study from our group (not published) and was submitted to MG-RAST (MG-RAST ID: 4539290.3 for sample P; 4539291.3 for sample E). The samples were collected in Arraial do Cabo (Rio de Janeiro State)—Brazil, at Praia dos Anjos Bay (−22°58′31.33′′, −42°0′46.84′′). No specific permits were required for the described field studies. The water was collected from surface (<2 m) and filtered first through 0.8 μm membranes (aiming to hold eukaryotes and particle-associated prokaryotes—named Sample E) and then through 0.22 μm membranes (aiming to hold free living prokaryotes—named sample P) using a vacuum filtration system. The DNA from both samples were extractedusing the kit DNA isolation kit for water (EPICENTRE) and sequenced by 454 FLX+ (ROCHE).

### 3.2. Metagenomic Reads Assembly

The metagenomic reads from both samples were assembled using CAP3 [29] with default parameters. The contigs and singlets were concatenated and the METAGENMARK [36] was used to extract the metagenomic Open Reading Frames (ORFs) and to translate those ORFs to protein sequences using the Transeq program from the EMBOSS package [48].

### 3.3. Screening for Genes of NRPS C Domain and Type I PKS KS Domains

A HMM profile (pHMM) approach was used to screen the genes of type I PKS (KS domains) and NRPS (C domains). The pipeline used in this work was adapted from a previous work developed by Dumaresq *et al.* [49]. The KS domains of type I PKS (modular and iterative) were obtained from MAPSIDB [50]. The NRPS C domains were obtained from NRPSDB [51,52]. For each domain, a multiple alignment was generated by MAFFT [53] and a HMM profile was built using the hmmbuild software from the HMMER v3.0 [41] package. Those profiles (PKS-pHMM and NRPS-pHMM) were then used to screen the translated ORFs of both samples from Arraial do Cabo using hmmsearch with *E*-value cutoff 0.1.

The translated ORFs that showed hits with PKS-pHMM and NRPS-pHMM were extracted from our metagenome dataset using FASTACMD (from BLAST 2.2.21 package [54]). A reverse search of the extracted translated ORFs against the PKS-pHMM and NRPS-pHMM was conducted using hmmscan (*E*-value cutoff 0.1), in order to classify the type of PKS from metagenomic sequences. In order to confirm and validate the results, all the extracted translated ORFs similar to profiles were submitted to BLASTP 2.2.21 (*E*-value cutoff e^−5^) against RefSeq protein database release 61. The annotation of the best five hits of each environmental sequence was used to annotate them. Finally, the confirmed KS and C domain sequences were submitted to Natural Product Domain Seeker—NapDoS [40,55] in order to classify the domains and to carry out phylogenetic analysis.

## 4. Conclusions

In this work, two fractions of the seawater metagenome from Praia dos Anjos (Angel’s Beach) a coastal environment, affected by upwelling, were screened for PKS and NRPS domains.

Screening of the metagenome revealed 84 KS domain ORFs and 46 C domain ORFs (from PKS and NRPS). These sequences were manually verified and classified by NapDoS system and BLAST, showing a close enough similarity to curated sequences to infer annotation for these sequences. However, the degree of divergence suggests that they are probably new alleles. Based on these results we will now prepare an environmental DNA (fosmid) library from which to clone full sequences of PKS and NRPS, in order to conduct functional and sequence screening, using the sequences generated in this study as probes. In addition, a time-series study may be conducted in the future, to better understand the main differences between the microbial communities from each season in this region.

## References

[1] Walker J.J., Spear J.R., Pace N.R. (2005). Geobiology of a microbial endolithic community in the Yellowstone geothermal environment. Nature.

[2] Uchiyama T., Abe T., Ikemura T., Watanabe K. (2005). Substrate-induced gene-expression screening of environmental metagenome libraries for isolation of catabolic genes. Nat. Biotechnol..

[3] Gregoracci G.B., Nascimento J.R., Cabral A.S., Paranhos R., Valentin J.L., Thompson C.C., Thompson F.L. (2012). Structuring of bacterioplankton diversity in a large tropical bay. PLoS ONE.

[4] Trindade-Silva A.E., Rua C., Silva G.G.Z., Dutilh B.E., Moreira A.P.B., Edwards R.A., Hajdu E., Lobo-Hajdu G., Vasconcelos A.T., Berlinck R.G. (2012). Taxonomic and functional microbial signatures of the endemic marine sponge *Arenosclera brasiliensis*. PLoS ONE.

[5] Cury J.C., Araujo F.V., Coelho-Souza S.A., Peixoto R.S., Oliveira J.A.L., Santos H.F., Dávila A.M., Rosado A.S. (2011). Microbial diversity of a Brazilian coastal region influenced by an upwelling system and anthropogenic activity. PLoS ONE.

[6] Ferreira C.E.L., Gonçalves J.E.A., Coutinho R. (2006). Ship hulls and oil platforms as potential vectors to marine species introduction. J. Coast. Res..

[7] López M.S., Coutinho R. (2010). Positive interaction between the native macroalgae *Sargassum* sp. and the exotic bivalve Isognomon bicolor?. Braz. J. Oceanogr..

[8] Coelho-Souza S.A., Pereira G.C., Coutinho R., Guimarães J.R. (2013). Yearly variation of bacterial production in the Arraial do Cabo protection area (Cabo Frio upwelling region): An evidence of anthropogenic pressure. Braz. J. Microbiol..

[9] Liu B., Gibbons T., Ghodsi M., Treangen T., Pop M. (2011). Accurate and fast estimation of taxonomic profiles from metagenomic shotgun sequences. BMC Genom..

[10] Jiang C.-J., Hao Z.-Y., Zeng R., Shen P.-H., Li J.-F., Wu B. (2011). Characterization of a novel serine protease inhibitor gene from a marine metagenome. Mar. Drugs.

[11] Gokhale R.S., Sankaranarayanan R., Mohanty D. (2007). Versatility of polyketide synthases in generating metabolic diversity. Curr. Opin. Struct. Biol..

[12] Koglin A., Walsh C.T. (2009). Structural insights into nonribosomal peptide enzymatic assembly lines. Nat. Prod. Rep..

[13] Lal R., Kumari R., Kaur H., Khanna R., Dhingra N., Tuteja D. (2000). Regulation and manipulation of the gene clusters encoding type-I PKSs. Trends Biotechnol..

[14] Cane D.E. (1998). Harnessing the Biosynthetic Code: Combinations, Permutations, and Mutations. Science.

[15] Sun W., Peng C., Zhao Y., Li Z. (2012). Functional gene-guided discovery of type II polyketides from culturable actinomycetes associated with soft coral *Scleronephthya* sp.. PLoS ONE.

[16] Castoe T.A., Stephens T., Noonan B.P., Calestani C. (2007). A novel group of type I polyketide synthases (PKS) in animals and the complex phylogenomics of PKSs. Gene.

[17] Silva-Stenico M.E., Silva C.S.P., Lorenzi A.S., Shishido T.K., Etchegaray A., Lira S.P., Moraes L.A., Fiore M.F. (2011). Non-ribosomal peptides produced by Brazilian cyanobacterial isolates with antimicrobial activity. Microbiol. Res..

[18] King G.M., Smith C.B., Tolar B., Hollibaugh J.T. (2012). Analysis of composition and structure of coastal to mesopelagic bacterioplanktoncommunities in the northern gulf of Mexico. Front. Microbiol..

[19] Jamieson R.E., Rogers A.D., Billett D.S.M., Smale D.A., Pearce D.A. (2012). Patterns of marine bacterioplankton biodiversity in the surface waters of the Scotia Arc, Southern Ocean. FEMS Microbiol. Ecol..

[20] Lau S.C.K., Zhang R., Brodie E.L., Piceno Y.M., Andersen G., Liu W.T. (2013). Biogeography of bacterioplankton in the tropical seawaters of Singapore. FEMS Microbiol. Ecol..

[21] Desriac F., Jégou C., Balnois E., Brillet B., Le Chevalier P., Fleury Y. (2013). Antimicrobial peptides from marine proteobacteria. Mar. Drugs.

[22] Graça A.P., Bondoso J., Gaspar H., Xavier J.R., Monteiro M.C., de la Cruz M., Oves-Costales D., Vicente F., Lage O.M. (2013). Antimicrobial activity of heterotrophic bacterial communities from the marine sponge Erylus discophorus (Astrophorida, Geodiidae). PLoS ONE.

[23] Schneemann I., Nagel K., Kajahn I., Labes A., Wiese J., Imhoff J.F. (2010). Comprehensive investigation of marine actinobacteria associated with the sponge *Halichondria panicea*. Appl. Environ. Microbiol..

[24] Grossart H.-P., Schlingloff A., Bernhard M., Simon M., Brinkhoff T. (2004). Antagonistic activity of bacteria isolated from organic aggregates of the German Wadden Sea. FEMS Microbiol. Ecol..

[25] Martens T., Gram L., Grossart H.-P., Kessler D., Muller R., Simon M., Wenzel S.C., Brinkhoff T. (2007). Bacteria of the Roseobacter clade show potential for secondary metabolite production. Microb. Ecol..

[26] Milne P.J., Hunt A.L., Rostoll K., van der Walt J.J., Graz C.J. (1998). The biological activity of selected cyclic dipeptides. J. Pharm. Pharmacol..

[27] Slightom R.N., Buchan A. (2009). Surface colonization by marine roseobacters: Integrating genotype and phenotype. Appl. Environ. Microbiol..

[28] Cude W.N., Mooney J., Tavanaei A.A., Hadden M.K., Frank A.M., Gulvik C.A., May A.L., Buchan A. (2012). Production of the antimicrobial secondary metabolite indigoidine contributes to competitive surface colonization by the marine roseobacter *Phaeobacter* sp. strain Y4I. Appl. Environ. Microbiol..

[29] Huang X., Madan A. (1999). CAP3: A DNA sequence assembly program. Genome Res..

[30] Lai B., Ding R., Li Y., Duan L., Zhu H. (2012). A *de novo* metagenomic assembly program forshotgun DNA reads. Bioinformatics.

[31] Namiki T., Hachiya T., Tanaka H., Sakakibara Y. (2012). MetaVelvet: An extension of Velvet assembler to *de novo* metagenome assembly from short sequence reads. Nucleic Acids Res..

[32] Afiahayati, Sato K., Sakakibara Y. (2013). An extended genovo metagenomic assembler by incorporating paired-end information. Peer J..

[33] Reddy R.M., Mohammed M.H., Mande S.S. (2014). MetaCAA: A clustering-aided methodology for efficient assembly of metagenomic datasets. Genomics.

[34] Mavromatis K., Ivanova N., Barry K., Shapiro H., Goltsman E., McHardy A.C., Rigoutsos I., Salamov A., Korzeniewski F., Land M. (2007). Use of simulated data sets to evaluate the fidelity of metagenomic processing methods. Nat. Methods.

[35] Pignatelli M., Moya A. (2011). Evaluating the fidelity of *de novo* short read metagenomic assembly using simulated data. PLoS ONE.

[36] Zhu W., Lomsadze A., Borodovsky M. (2010). Ab initio gene identification in metagenomic sequences. Nucleic Acids Res..

[37] Kennedy J., Codling C.E., Jones B.V., Dobson A.D.W., Marchesi J.R. (2008). Diversity of microbes associated with the marine sponge, *Haliclona simulans*, isolated from Irish waters and identification of polyketide synthase genes from the sponge metagenome. Environ. Microbiol..

[38] Trindade-Silva A.E., Rua C.P.J., Andrade B.G.N., Vicente A.C.P., Silva G.G.Z., Berlinck R.G., Thompson F.L. (2013). Polyketide synthase gene diversity within the microbiome of the sponge *Arenosclera brasiliensis*, endemic to the Southern Atlantic Ocean. Appl. Environ. Microbiol..

[39] Foerstner K.U., Doerks T., Creevey C.J., Doerks A., Bork P. (2008). A Computational screen for type I polyketide synthases in metagenomics shotgun data. PLoS ONE.

[40] Ziemert N., Podell S., Penn K., Badger J.H., Allen E., Jensen P.R. (2012). The natural product domain seeker NaPDoS: A Phylogeny based bioinformatic tool to classify secondary metabolite gene diversity. PLoS ONE.

[41] Eddy S.R. (2011). Accelerated profile HMM searches. PLoS Comput. Biol..

[42] Jenke-Kodama H., Sandmann A., Muller R., Dittmann E. (2005). Evolutionary implications of bacterial polyketide synthases. Mol. Biol. Evol..

[43] Tringe S.G., von Mering C., Kobayashi A., Salamov A.A., Chen K., Chang H.W., Podar M., Short J.M., Mathur E.J., Detter J.C. (2005). Comparative metagenomics of microbial communities. Science.

[44] Handelsman J., Rondon M.R., Brady S.F., Clardy J., Goodman R.M. (1998). Molecular biological access to the chemistry of unknown soil microbes: A new frontier for natural products. Chem. Biol..

[45] Venter J.C., Remington K., Heidelberg J.F., Halpern A.L., Rusch D., Eisen J.A., Wu D., Paulsen I., Nelson K.E., Nelson W. (2004). Environmental genome shotgun sequencing of the Sargasso Sea. Science.

[46] Fisch K.M. (2013). Biosynthesis of natural products by microbial iterative hybrid PKS–NRPS. RSC Adv..

[47] Slattery M., Rajbhandari I., Wesson K. (2001). Competition-mediated antibiotic induction in the marine bacterium *Streptomyces tenjimariensis*. Microb. Ecol..

[48] Rice P., Longden I., Bleasby A. (2000). EMBOSS: The European molecular biology open software suite. Trends Genet..

[49] Romão-Dumaresq A.S., Fróes A.M., Cuadrat R.R.C., Silva F.P., Dávila A.M.R. (2014). Towards a comprehensive search of putative chitinases sequences in environmental metagenomic databases. Nat. Sci..

[50] MAPSIDB. http://gate.smallsoft.co.kr:8080/pks/mapsidb.

[51] NRPSDB. http://linux1.nii.res.in/~zeeshan/webpages/home.html.

[52] Ansari M.Z., Yadav G., Gokhale R.S., Mohanty D. (2004). NRPS-PKS: A knowledge-based resource for analysis of NRPS/PKS megasynthases. Nucleic Acids Res..

[53] Katoh K., Misawa K., Kuma K., Miyata T. (2002). MAFFT: A novel method for rapid multiple sequence alignment based on fast Fourier transform. Nucleic Acids Res..

[54] Altschul S.F., Gish W., Miller W., Myers E.W., Lipman D.J. (1990). Basic local alignment search tool. J. Mol. Biol..

[55] NapDOS. http://npdomainseeker.ucsd.edu/.

